# Corrosion behavior and microstructure of Al–10Zn alloy with nano CuO addition

**DOI:** 10.1038/s41598-023-39515-6

**Published:** 2023-08-08

**Authors:** Eman AbdElRhiem, Saad G. Mohamed, Yosry F. Barakat, M. M. Mostafa, R. H. Nada, Shereen M. Abdelaziz

**Affiliations:** 1 Mining and Metallurgy Engineering Department, Tabbin Institute for Metallurgical Studies (TIMS), Tabbin, Helwan 109, Cairo, 11421 Egypt; 2 Physics Department, Faculty of Education, Ain Shams University, Heliopolis 11771, Roxy, P.O. Box 5101, Cairo, Egypt

**Keywords:** Metamaterials, Composites, Materials science, Metals and alloys

## Abstract

The present study explores the preparation of Al–10wt.%Zn alloy by the casting process. Nano CuO was prepared by the Co-precipitation method. The effect of adding nanostructure of (1wt.% CuO) to Al–10Zn alloy was studied the corrosion effects as-cast and with different aging temperatures (423, 443, and 463 K) for 2 h in 3.5% NaCl aqueous solution after homogenized for 2 h at 500 K at room temperature. Electrochemical measurements (OCP, Tafel, and EIS) were performed to determine the corrosion rate (C.R.) and corrosion current density (I_corr._) to find out corrosion behavior. In addition, microstructures of Al–10Zn and Al–10Zn–1CuO were observed using a scanning electron microscope, EDX mapping, and the optical microscope to investigate the effect of the nanoparticle’s addition before and after aging and the corrosion test. The average crystal size and the dislocation density were calculated from the XRD pattern. The results show that the appropriate addition of CuO nanoparticles can refine the Al–10Zn alloy and shift the Al–10Zn alloy to a more noble direction.

## Introduction

Aluminum (Al) and its alloys have recently been employed extensively in modern engineering applications due to its high strength and lightweight^1,2^. Moreover, their hardness, low wear, and chemical resistance limit their use in various automotive, construction, and aerospace applications^3–6^. Pure Al has weak mechanical characteristics in engineering applications, whereas alloying and heat treatment can improve it. The proper Al alloys must be chosen for required applications considering their specific rigidity, thermic conductivity, low density, strength, formability, weldability, workability, ductility, wear, and corrosion resistance^7^.

There are more and more applications for cast alloys made from Al and Zinc (Zn)-based matrices, and their production is rising globally^8^.

The primary purpose for concentrating on Al–Zn alloys is that adding Zn causes value addition, improves the matrix’s homogeneity, and enhances Al alloys properties^9^. Zn has a high solubility in the Al matrix; adding Zn causes a low lattice distortion, which almost has no impact on the alloy’s formability^10^. Al–Zn alloys have high strength, ductility, heat treatability, excellent hot workability/formability, and good welding properties^11,12^. Al–Zn alloys also have a considerable impact on their microstructure, being a fine-grained industrial alloy used to create high-strength corrosion-proof (verification) structures for aircraft, ships, and vehicle buildings^13^. Hence, it is necessary to constantly support the Al matrix with suitable Ceramic nano particulate reinforced ceramics such as CuO, TiO_2_, SiC, SiO_2_, B_4_C, and Al_2_O_3_^14,15^_._ They are considered the best option for Al as a matrix base metal because they impart high strength and resistance to wear and corrosion^16^. Ceramic particles play a role in increasing mechanical strength by acting as a nucleation site for solidification, allowing the grain size to be finer. Alloying elements’ function is to form a solid solution of Al alloy, which causes grain size refinement. At the same time, the role of alloying elements is to develop a solid solution of Al alloy which causes grain size refinement. There are different ways to make Al alloys’ nanocomposites, such as stir casting, which mainly works in manufacturing the composites as it produces composites with uniform reinforcement distribution^6,17–19^.

CuO is one of the best choices for Al matrix nanocomposite because it has many advantages; CuO addition in Al matrix material improves corrosion resistance, stability, stiffness structural applications, particularly for aerospace and automobile engineering, and thermal properties^20^. CuO was chosen in this study for various reasons, including; commercially, CuO particles were used to create Al-based composites due to their superior mechanical and physical properties^21^. Low-cost, widely available. CuO has received much research attention due to its many valuable uses in electrical equipment, including Solar cells, highly hydrophobic surfaces, and gas detection sensors^22^. Cu addition lowers the melting point and may cause the creation of the Al_2_Cu phase, which increases the Al matrix’s tensile strength^21^. Nano-copper oxide effectively reduces friction and prevents wear on machinery parts because of its hardness^23^. Al and CuO phases have differing structures and stresses, which makes adding CuO nanoparticles to the Al matrix advantageous. At the point where the Al matrix and CuO reinforcement particles meet, it creates dislocation. The strength of the Al matrix, which is connected to the static dislocations generated during the work hardening (aging) process, is increased due to the created dislocations’ increased surface area and increased grain refinement, which improves corrosion resistance^24^.

To study the effect of Al–CuO composite on Al sheets, Hamed et al*.* prepared Al sheets and Al–CuO composite by accumulative roll bonding, adding 0.5 vol.% CuO to pure Al. They reported that the Al–CuO composite showed that microhardness and tensile strength analysis was higher than the pure Al; reinforcing in particles acts like pins on Al^24^. Usually, heat treatment increases formability. Nonetheless, depending on the heat treatment’s temperature and time, it can result in recovery, recrystallization, or grain growth. Strength and toughness are consequently reduced. Increased work hardening and improved grain refinement are caused by the addition of nanostructure^24^. Wang et al*.*^25^ recently investigated the electrochemical behavior of an Al–Zn alloy. The authors examined the electrochemical corrosion test on Al–20Zn–0.2In alloy and discovered a reduced corrosion rate. Zhao et al.^26^ used electron back-scattered diffraction to study the microstructural evolutions of an Al–Zn–Mg–Cu alloy processed by multiaxial forging in various directions (z-axis to the x-axis to the y-axis to z-axis to again x-axis), while rotating the sample 90° each time. All the samples were heating at 573 K and suffered various degrees of deformation. As the number of multiaxial deformations increased, the formed precipitate decreased. Due to precipitates pinning grain development, grain size decreased as multiaxial deformations increased. Hu et al*.*^27^ investigated how Ce affected the Al–Zn–Mg alloy’s microstructural changes and corrosion behavior. High-purity Al, Zn, Mg, and Ce ingots and rods were combined to prepare the alloy through the casting process. The casting process was carried out at 1023 K, the cast samples were heated for 4 h at 693 K, and then the specimens were extruded at 733 K using an 11:1 extrusion ratio. The microstructural and stress corrosion cracking was performed to evaluate the alloy performances. The addition of Ce increased homogenous microstructure and corrosion resistance. Pan et al*.*^28^ studied two nano-treated AA7075 alloys (Al–Zn–Mg–Cu) cast and extruded were tested for corrosion behavior and microstructure. The results showed that the nano-treated AA7075 (TiC and TiB_2_) have higher hardness considering the refine grain size. At the same time, the localized electrons at the matrix-nanoparticle interfaces cause reduced electrical conductivity, which might minimize corrosion reactivity. AA7075 nano-treated alloys may exhibit improved corrosion resistance for either of these reasons. Simoes et al.^29^ studied the microstructural and mechanical characteristics of Al matrix composites produced by the powder metallurgy technique and reinforced with varying amounts of CNTs (0, 0.5, 0.75, 1, and 1.5 vol). The study’s findings demonstrated that nanocomposites with a 1 vol% CNT reinforcement increase their strength due to their strong dispersion efficiency. AbdElRhiem et al*.*^30^ studied the Effects of adding TiO_2_, CuO, and SiO_2_ nanoparticles on Al–Zn alloy's microstructure and mechanical properties. The study found nanoparticle addition resulted in grain refinement and hardening of the Al–Zn alloy.

The above discussion shows insufficient corrosion behavior, and pit formation data are available. This study has highlighted the significance of Al–10wt.%Zn alloy and the effect of nano 1wt.%CuO addition as a reinforcement to improve and enhance the performance of the alloy (electrochemical behavior and microstructure). 1CuO nanostructure was added to Al–10Zn by the mechanical dispersion method. Electrochemical measurements were performed at room temperature to determine the corrosion behavior before and after different aging temperatures (423, 443, and 463 K) for 2 h.

## Materials and methods

### Nano CuO preparation

Copper oxide nanostructure was prepared by the co-precipitation method using copper chloride (CuCl_2_) as a copper source and NaOH as a precipitating agent. Distilled water was used to filter and wash the precipitate. Then the precipitate was grinded and overnight dried at 373 K. Finally, the powder was calcinated at 773 K for 2 h.

### Samples fabrication and treatment

In the present study, the base matrix and reinforcement particles are Al–10wt.% Zn and CuO nanostructure addition (Al–10wt.% Zn–1wt.%CuO). Using the raw materials, an Al–10wt.% Zn alloy was created. At 1023 K, Al and Zn were melted and mixed in high-purity graphite crucibles in an Ar atmosphere. The molten alloy was poured into a steel mould to make the cold-cast ingots. Ten millimeter-diameter rod-shaped samples were collected. In addition, reinforcement particles were created by mechanically distributing 1% CuO nanostructure into an Al–10%Zn alloy. The samples were remelted in a vacuum arc furnace at 1050 K under a high-quality argon flow protection and then cast into stainless steel molds to achieve a homogeneous composition, obtaining 10 mm diameter rod-like samples. These rod-shaped samples were swaged and then cold-pulled into sheets that were 1 mm thick. After solidification, the ingots were homogenized for 2 h at 500 K before being cooled slowly to ambient temperature.

The samples were then aged for 2 h at three different temperatures (423, 443, and 463 K) before being quenched in cooled water to room temperature to preserve the structure created at these aging temperatures.

**Figure 1:**
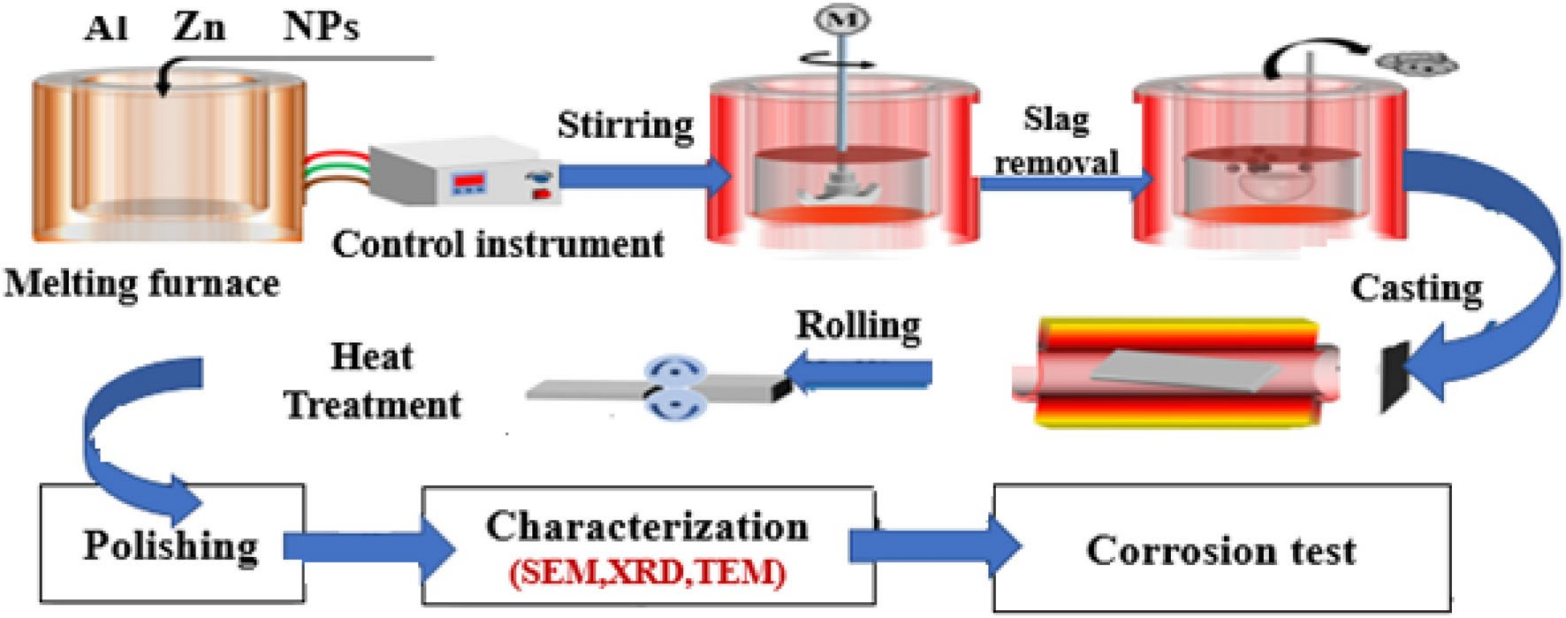
Schematic diagram of the fabrication process for the Al–10Zn and CuO addition nanostructure.

Table 1 displays the investigated samples’ chemical composition (in weight percent). The schematic diagram (Fig. 1) shows the preparation steps of the matrix Al–10wt.% Zn and Al–10wt.% Zn–1wt.%CuO.

**Table 1:** Chemical composition of Al–10Zn and Al–10Zn–1CuO by weight percentage.

Chemical composition	Symbol	Al	Zn	Si	Cu	O
Al–10wt.%Zn alloy	**A**_**1**_	Bal	9.58	–	–	–
Al–10wt.%Zn–1wt.% CuO	**A**_**2**_	Bal	9.45	–	0.66	0.33

### Material characterization

Mechanical grinding and polishing were used on the surface samples for the microstructure investigations. Keller’s reagent etching was then applied to reveal their grain boundaries and orientations. (2.5 ml HNO_3_, 1.5 ml HCl, 1 ml HF, and 95 ml distilled water).

The morphology of the obtained samples was investigated using emission scanning electron microscopy (FE-SEM) JOEL, JSM-6700F equipped with an energy dispersive X-ray spectroscopy (EDS), optical microscope (OM) LECO LX 31 with a magnification of up to 500, and transmission electron microscopy (TEM, JEOL JEM_2100). The phase analysis was performed using an X-ray diffractometer (Bruker AXSD8 X-ray diffractometer, ADVANCE, Germany) with Cu Kα radiation at λ = 1.5406 Å.

### Corrosion testing

The corrosion test of the samples was studied using a three-electrode system according to ASTM G59-97 using an electrochemical testing station (Origaflex-OGF01A-Origalys, France) at room temperature. Before testing, the samples were subjected to mechanical polishing with 1 cm × 1 cm dimensions. The samples were cleaned with deionized water, then ultrasonically cleaned in acetone and dried. Then, the corrosion test was formed in a solution of NaCl 3.5% concentration. The Pt sheet served as an auxiliary electrode, and an Ag/AgCl was used as the reference electrode.

The electrochemical measurements were done in two steps: (i) obtaining a time dependence of potential over a period of 30 min that allowed for the measurement of the static potential; and (ii) obtaining the curves “corrosion current density (Id)–potential (E) in the potential between −300 and 300 mV with respect to E_corr_, with the scan rate of 2 mV/s.

In addition, use the Tafel extrapolation method to get the Tafel slopes. The electrochemical impedance spectroscopy (EIS) measurements were performed at a frequency range of 0.1 Hz to 100 kHz at the open circuit potential with an AC sine wave amplitude of 10 mV (ASTM G106-89).

## Results and discussion

### Microstructural investigations

#### CuO nanostructure

**Figure 2:**
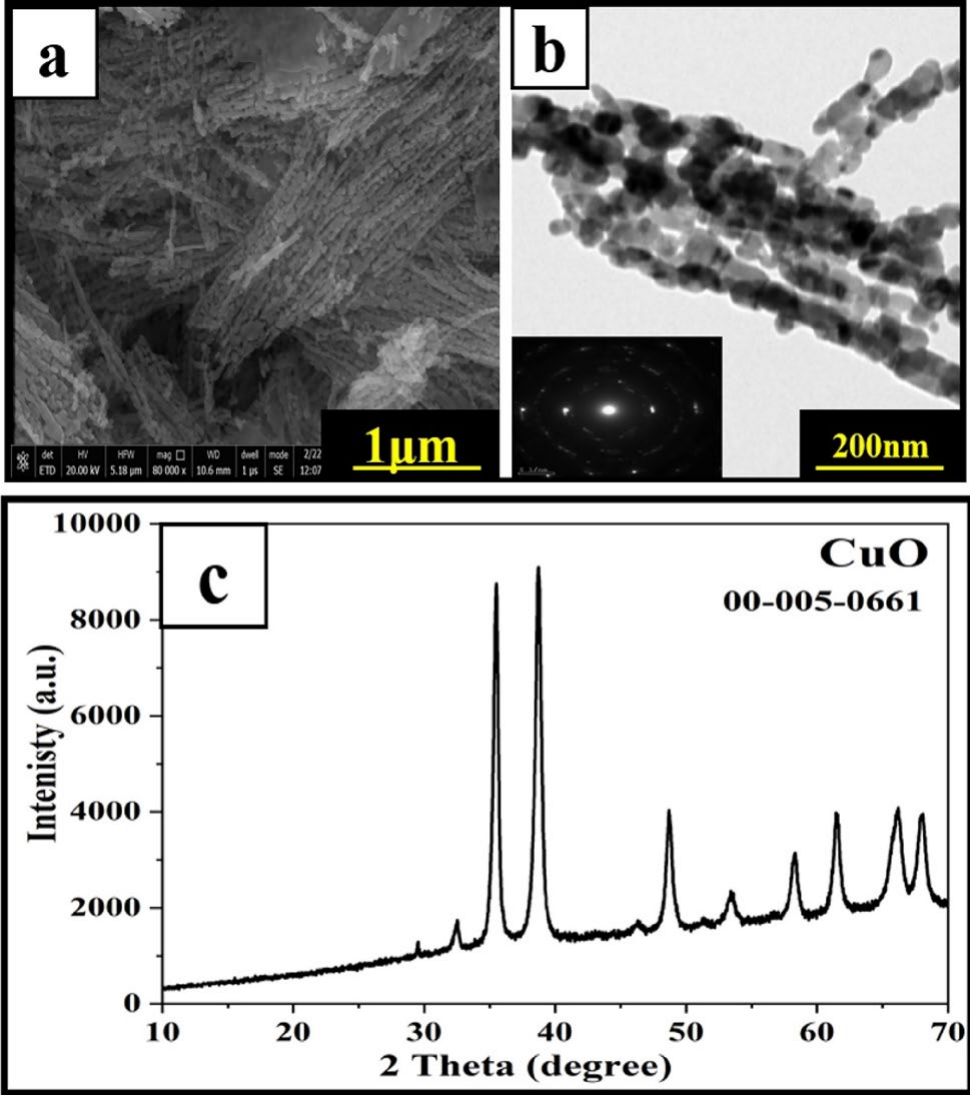
SEM image (**a**) and TEM image (**b**) of the prepared CuO nanorods and (**c**) XRD pattern of the synthesized nano CuO.

Figure 2 shows the SEM images of the CuO nanostructure. Nanorod morphology with porous nature can be observed in both SEM (Fig. 2a) and TEM images (Fig. 2b), indicating the successful preparation of the CuO nanostructure.

The XRD pattern of the prepared copper oxide (Fig. 2c) shows peaks at 2θ equal to 32.5°, 35.6°, 38.7°, 38.9°, 46.3°, 48.8°, 51.3°, 53.4°, 58.3°, 61.6°, 66.2° and 68.1° with monoclinic phase^31^ (card no. 00-005-0661) which confirms the preparation of pure CuO. The XRD shows sharp peaks, revealing the high crystallinity of the prepared CuO^22^.

#### Microstructure of the samples

**Figure 3:**
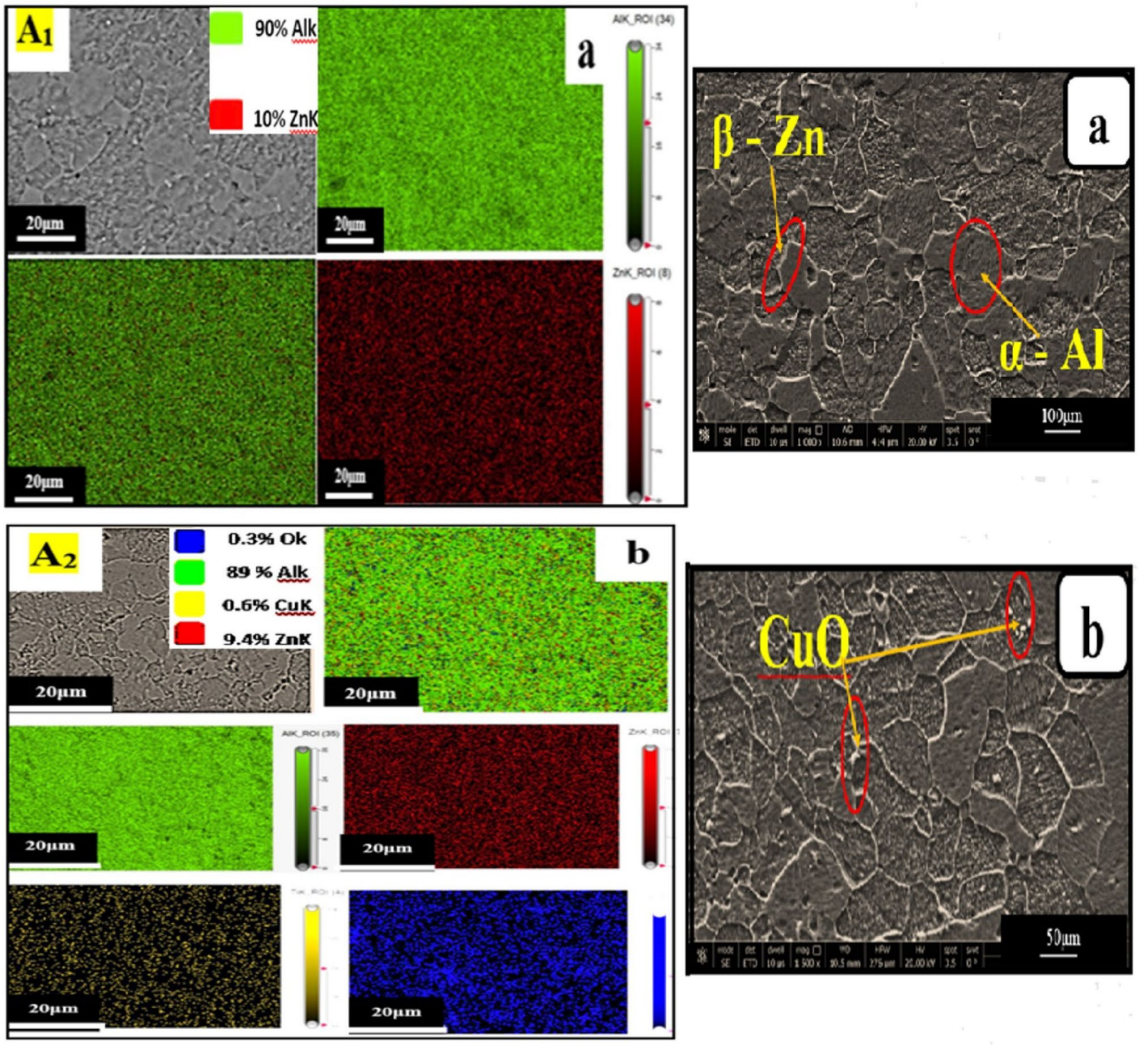
EDX mapping and SEM images of a homogeneous microstructure of (**a**) **A**_**1**_ and (**b**)** A**_**2**_.

Figure 3 shows The EDX mapping and SEM images of a homogeneous microstructure of **A**_**1**_ and **A**_**2**_, showing two metallurgical phases: an α-Al matrix and β-Zn phase, where the Zn single large particles nucleate at the grain boundary with white color, and the grey color represents Al, as shown in (Fig. 3a). When added to the base alloy, the CuO nanostructure represented the accumulated white particles, as shown in (Fig. 3b). The EDX mapping analysis confirms the chemical compositions of samples **A**_**1**_ and **A**_**2**_, respectively. In addition, Fig. 3 indicates that the CuO nanoparticles had an identical distribution (strong dispersion efficiency). The propensity of zinc to precipitate on the boundaries of the grains enhances the possibility of nanoparticle aggregation on the Zn particles that produce bright grey precipitates on grain boundaries, as shown in (Fig. 3a, b) during aging, which leads to an increase in their strength by acting as a nucleation site for solidification, following the grain size to be finner^6,17–19^.

**Figure 4:**
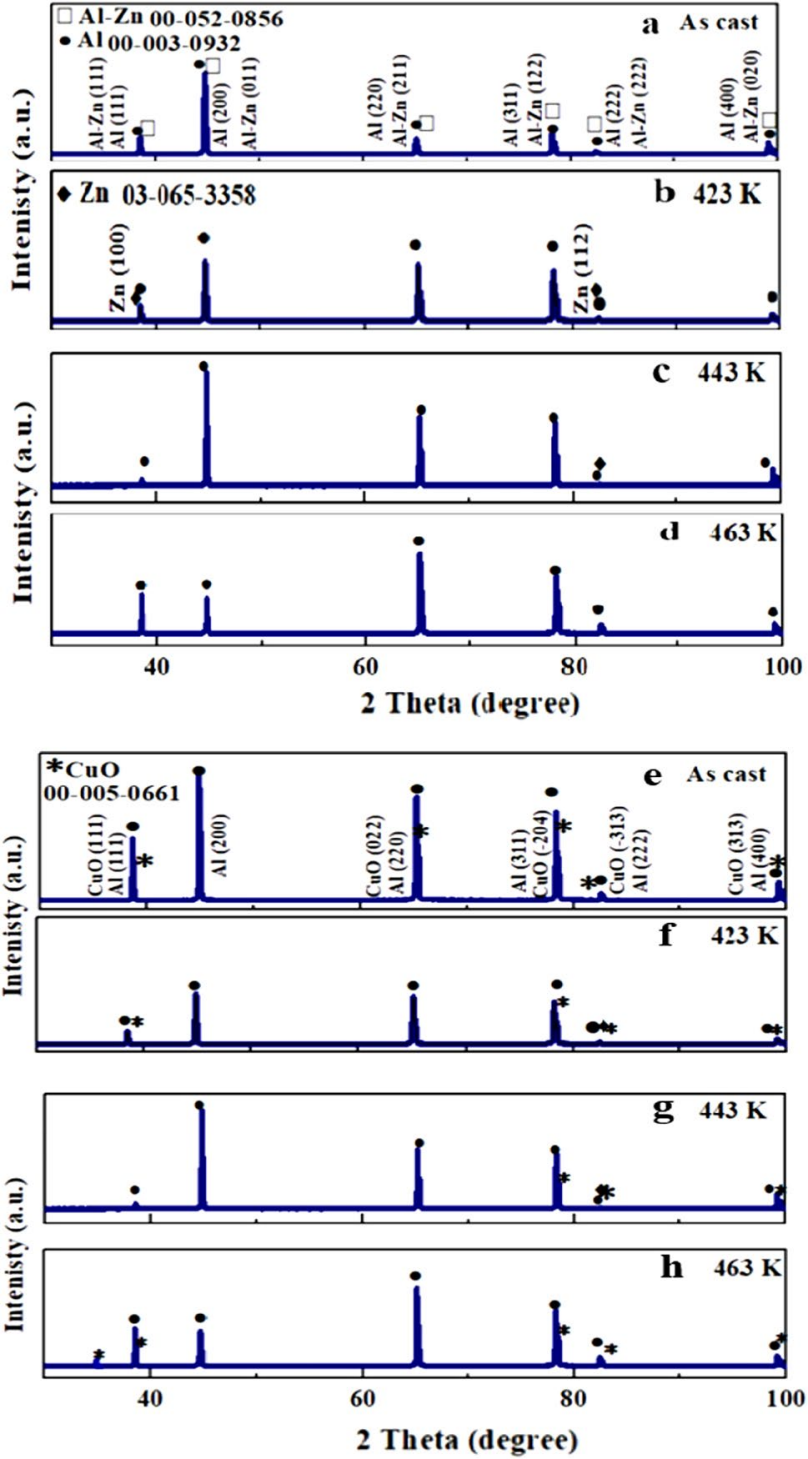
XRD patterns of (**a–d**) **A**_**1**_ and (**e–h**) **A**_**2**_ before and after aging.

Figure 4a–d, e–h show the XRD patterns of A1 and A2, as-cast and at different aging temperatures of 423 K, 443 K, and 463 K, respectively. The XRD pattern of the as-cast **A**_**1**_ alloy, shown in Fig. 4a, revealed an AlZn anorthic structure with a homogeneity distribution of a single phase, where Zn is completely dissolved in the Al matrix (reference code: 03-065-3358). The addition of nano-CuO to the Al–10Zn alloy appears to have formed CuO peaks, as shown in Fig. 4e (monoclinic CuO, reference code: 00-005-0661)^22^.

Figure 4b, c show the XRD patterns of **A**_**1**_ alloy after aging at 423 K and 443 K, revealing two phases, α-Al of fcc structure and β-Zn of hexagonal structure, with reference codes of 000030932 and 030653358, respectively. Furthermore, after the 443 K transition temperature, the diffraction peaks for Zn particles completely disappeared at 463 K, as shown in Fig. 4d, where Zn atoms are completely dissolved in the Al matrix and transferred into a single phase^32,33^.

The average crystal size (D) and the dislocation density (ρ) can be calculated from the XRD pattern by using Williamson-Hall’s calculation (Eq. 1)^24^.

1 $$\upbeta \frac{\mathrm{cos \theta }}{\uplambda }=(0.9/\mathrm{D})+\upvarepsilon (2\mathrm{ sin } \uptheta/\uplambda )$$

where θ is the Bragg angle, λ is the x-ray wavelength; β is the broadening of the diffraction line (FWHM). And the dislocation density was obtained for samples using Eq. (2)^34^.

2 $$\uprho \mathrm{d }= 14.4\frac{{\varepsilon }^{2} }{{b}^{2}}$$

**Figure 5:**
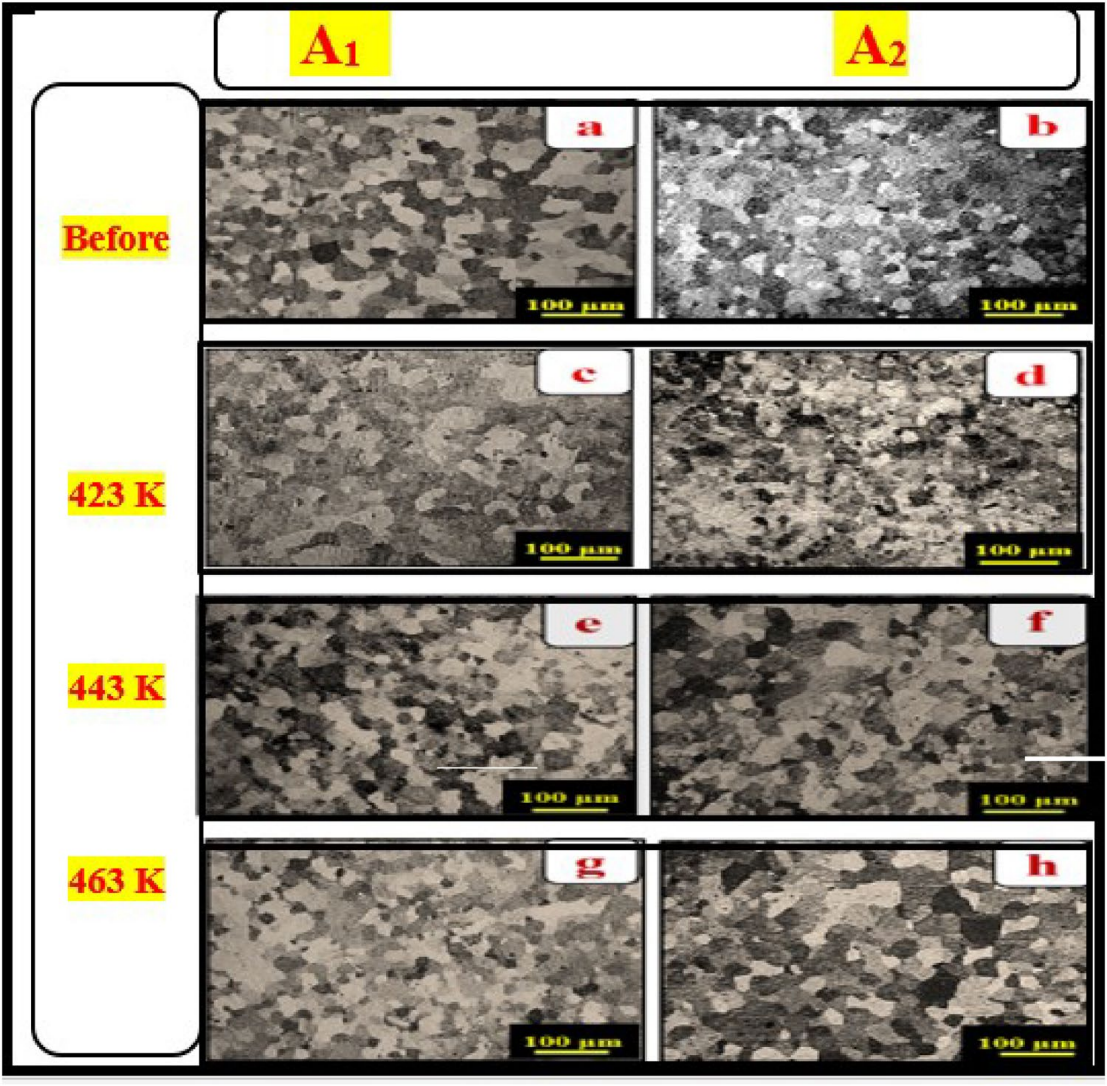
Optical images(OM) before and after aging **A**_**1**_ and **A**_**2**_ (scale bar 100 μm).

**Figure 6:**
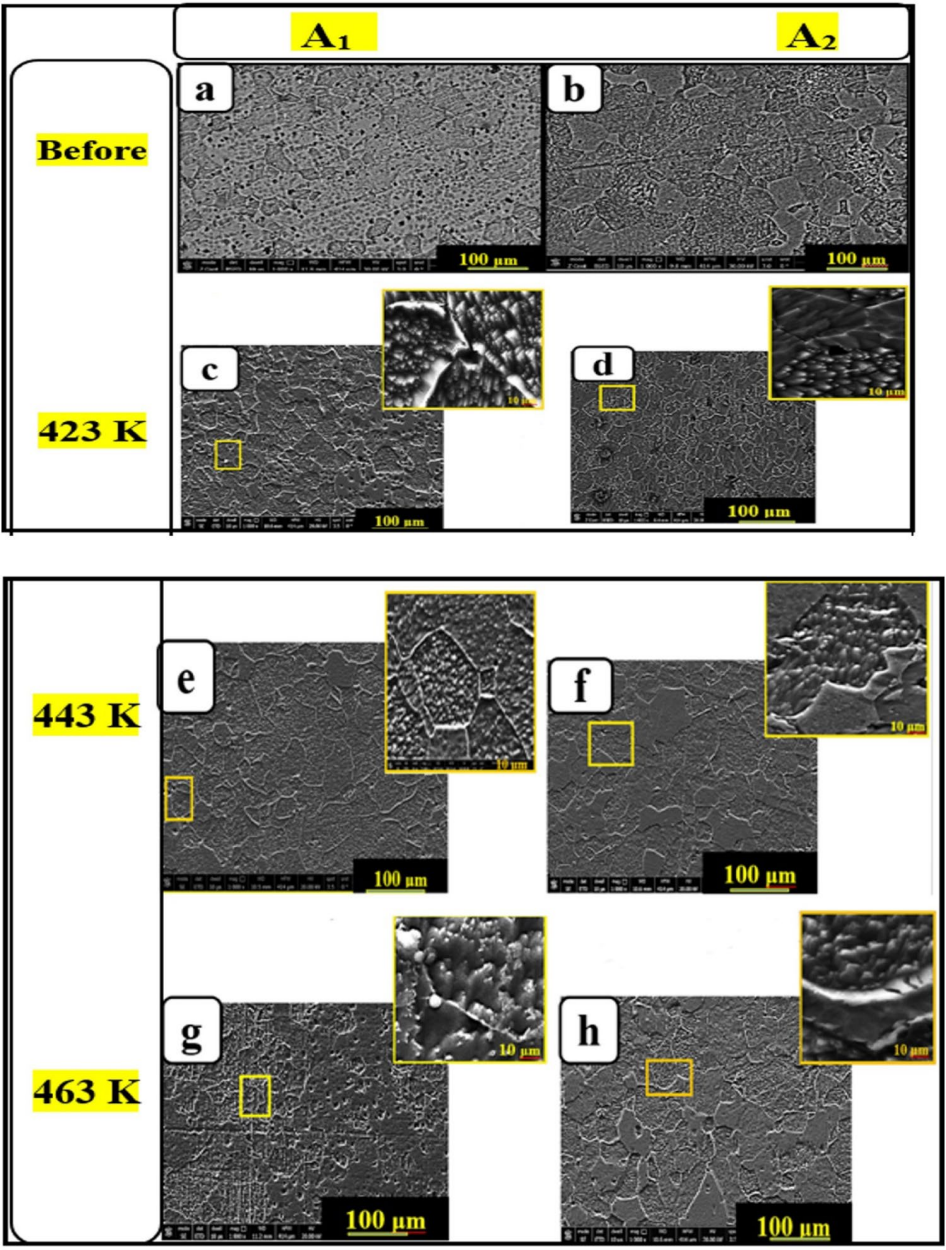
SEM images before and after aging temperatures of **A**_**1**_ and **A**_**2**_ at (scale bar 100 and 10 μm).

To understand the evolution of the polarization resistance, dislocation density was calculated, and detailed microstructure characterization was performed on all samples studied. The value of dislocation density obtained from XRD as a function of aging temperature is given in Table 2. The crystal size of **A**_**1**_ was decreased by adding nano CuO at different aging temperatures. Perhaps the formation of dislocations at the interface of the Al matrix and nano-CuO, as reinforcement particles, is caused by strain and different structural properties of the two phases, which improve microstructures and corrosion resistance^6,24^. The lattice strain and dislocation density decrease as the aging temperature rises, as discussed in Table 2 and as shown by XRD calculations, creating pinned mobile dislocations. This result led to significant refining in the grains of A_2_ due to their strong dispersion efficiency compared with A_1_, confirmed by OM and SEM images in Figs. 5 and 6_._ As a result, the strengthening characteristics of the nano-treated A2 increased and hardened than A_1_^24,30^.

**Table 2:** XRD analysis data crystal size and dislocation density.

Sample	Crystal size D (nm)				Dislocation density (ρ_d_/m^2^)			
	As-cast	423 K	443 K	463 K	As-cast	423 K	443 K	463 K
**A**_**1**_	714	290	243	279	1.96 × 10^13^	1.19 × 10^13^	1.68 × 10^13^	1.28 × 10^13^
**A**_**2**_	476	159	127	137	4.4 × 10^13^	3.9 × 10^13^	6.1 × 10^13^	5.3 × 10^13^

##### Optical microscope examinations (grain refinement)

Optical micrographs and SEM of **A**_**1**_ and **A**_**2**_ are shown in Figs. 5 and 6 before and after aging at different temperatures (423, 443, and 463 K), respectively. Grain size calculations using the Image J program showed that fine-refining is performed at all aging temperatures than as-cast one (Figs. 5 and 6a, b). **A**_**1**_ grains size is 168 μm while **A**_**2**_ is 176 μm. While after all aging temperatures, the **A**_**2**_ grains size was refined, as shown in Figs. 5 and 6d, f, h. Moreover, after 443 K, the grains’ size experienced more grain refinement than before. At 423 K, Figs. 5 and 6c, d, the mean grains size of **A**_**2**_ became smaller than **A**_**1**_ alloy, from 77 μm to 75 μm, but after the transformation point, the grains size of **A**_**2**_ became finer than **A**_**1**_ alloy, as shown in Figs.5 and 6e, f, from 102 μm to 63 μm at 443 K and then became more finner from 108 to 58 μm at 463 K, as shown in Figs. 5 and 6g, h. The mean grain size of **A**_**1**_ alloy and **A**_**2**_ was 113 and 93, respectively. Hence, aging resulted in modifying the structure of the aged samples as a consequence of the more uniform distribution of solute, especially for **A**_**2**_ than **A**_**1**_ alloy. Referring to dislocation density, as presented in Table 2, A2 caused an increase in the surface area compared with **A**_**1**_ alloy before and after aging temperatures, fine graining occurred and modifying the structure.

As a result, the grains of the **A**_**2**_ have different orientations than the grains of the **A**_**1**_ alloy, as shown in Figs. 5 and 6. The increasing number of dislocations density, as provided in Table 2 of **A**_**2**_, where the CuO nanostructure interface with metal matrix caused to increase in the surface areas. Adding CuO nanostructure created different structures of Al–Zn and CuO Phases, which caused the creation of dislocations in the interface of the Al matrix and reinforcement nano CuO^24^. The increased aging temperature caused a significant change in the microstructure besides the grain growth and affected the **A**_**2**_ grains size distribution^24^.

##### Corrosion characteristics of the samples

**Figure 7:**
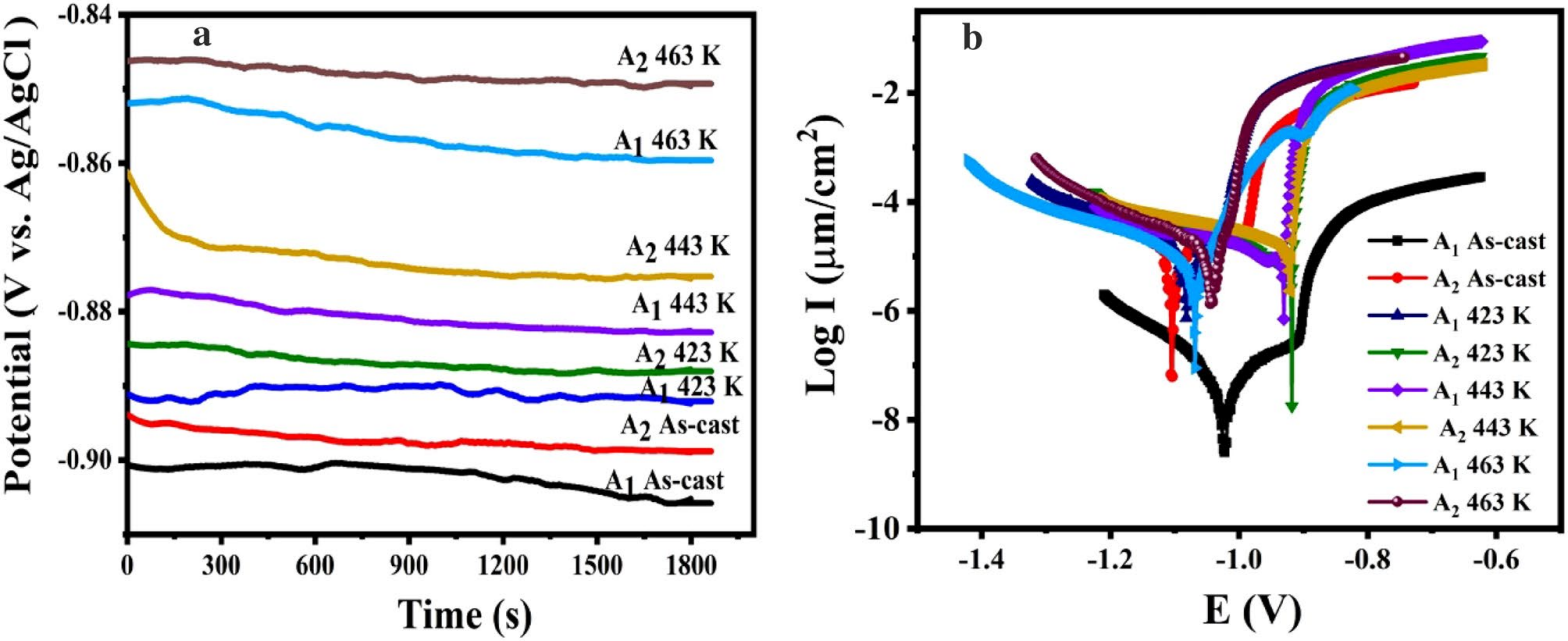
(**a**) OCP curves and (**b**) potentiodynamic polarization curves of the **A**_**1**_ and **A**_**2**_ in 3.5% NaCl solution before and after aging.

The open circuit potential (OCP) values of **A**_**1**_ and **A**_**2**_ were observed for 2000s while being as-cast and after being aged at different temperatures (423, 443, and 463 K) for 2 h before being submerged in a 3.5% NaCl solution at room temperature, as shown in Fig. 7 a. The OCP of both **A**_**1**_ and **A**_**2**_ increases steadily with increasing aging temperature during the OCP of immersion in NaCl. The potential of the **A**_**1**_ varies from −0.90065 to −0.96697 V vs. Ag/AgCl at aging temperature 423 K, while the potential of the **A**_**2**_ changes from −0.8959 to −0.96072 V vs. Ag/AgCl. The potential of the **A**_**1**_ shifts from −0.96697 to −0.96958 V vs. Ag/AgCl at aging temperature 443 K, while the potential of the **A**_**2**_ shifts from −0.96072 to −0.96141 V vs. Ag/AgCl. The potential of the **A**_**1**_ shifts from −0.96958 to −0.96974 V vs. Ag/AgCl at the aging temperature of 463 K, while the potential of the **A**_**2**_ changes from −0.96141 to −0.97876 V vs. Ag/AgCl. After being submerged in NaCl for longer periods (> 2000s), it is hypothesized that the OCP of the **A**_**1**_ and **A**_**2**_ may undergo an additional positive shift, with the potential shift of the **A**_**2**_ being expected to be greater than that of the **A**_**1**_. Al–10Zn was made better in terms of OCP when nano 1CuO was added, both as-cast and at all aged temperatures. The OCP for **A**_**1**_ and **A**_**2**_ that is optimally aged is at 463 K. OCP tests revealed the potential stability of A_2_ in 3.5% NaCl solution slowed down due to the addition of nano 1CuO and showed the influence of aging on positive potential shift by increasing temperature, which confirms the Tafel test. (Fig. 7b).

The potentiodynamic polarization was used to investigate the effect of adding 1 nano CuO to an Al–10Zn alloy on corrosion attack in a 3.5% NaCl solution at room temperature. The anodic and cathodic branch slopes are where the Tafel slopes are found. The corrosion parameters can be derived using these slopes.

The corrosion rate of the samples can be determined using the results of the polarization test. The following formula is used to determine the corrosion rate:

3 $$\mathrm{C}.\mathrm{R}. = \frac{0.0032 \times Icorr \times (M.W.)}{n \times d}$$

where n is the number of charge transfers that occur during the corrosion process, C.R. is the corrosion rate (mpy), I_corr_ is the current density of corrosion (A cm^−2^), M.W. is the molecular weight of the corroded material (g/mol), and **d** is the density of the corroded material (g cm^−3^)^35^.

The corrosion rates for the **A**_**1**_ and **A**_**2**_ in NaCl at room temperature, as determined by electrochemical measurements, are shown in Fig. 7b. The Tafel extrapolation measurements show the results of at least three tests for each condition of the as-cast and after-aged samples. It can be noted that the **A**_**2**_ corrosion rate is lower than the **A**_**1,**_ and also corrosion current density lowered over the brief testing period.

**Figure 8:**
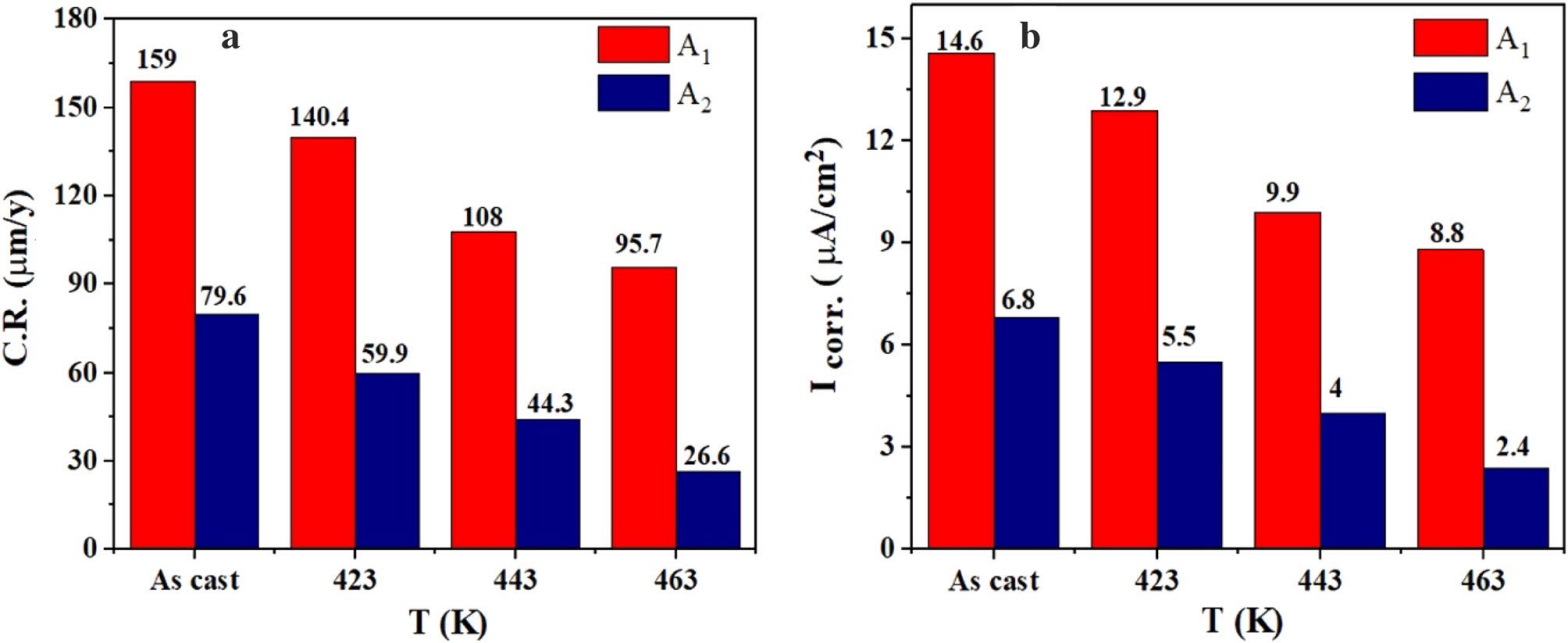
Relation between (**a**) C.R. and (**b**) I_corr._ vs. temperature of **A**_**1**_ and **A**_**2**_ before and after aging.

The Tafel plot yielded corrosion potential (E_corr_), I_corr_, and corrosion rate (C.R.). As shown in Fig. 8a, C.R. in as-cast samples decreased from 159 to 79.6 μm/y. C.R. of the aged sample at 423 K decreased from 140.4 to 59.9 μm/year. Also, C.R. reduced for the aged sample at 443 K from 108 to 44.3 μm/year, and the aged sample at 463 K from 95.7 to 26.6 μm/year. According to Fig. 8b, I_corr_ reduces for **A**_**1**_ alloy by increasing the aging temperature compared with the as-cast one. However, as shown in Fig. 8b, adding nano CuO to Al–10wt.%Zn alloy improved corrosion resistance (reduction of I_corr_) over **A**_**1**_. After all aging temperatures, **A**_**2**_ showed better corrosion resistance than **A**_**1**_ samples. By increasing the aging temperature, I_corr_ of **A**_**1**_ reduces. However, as shown in Fig. 8b, adding 1 nano CuO to **A**_**1**_ improved corrosion resistance (reduction of I_corr_) over **A**_**1**_. **A**_**2**_ demonstrated superior corrosion resistance to **A**_**1**_ samples for the as-cast and after-all aging temperatures. I_corr_ reduced in the as-cast samples from 14.6 to 6.8 μA/cm^2^, the sample at 423 K reduced from 12.9 to 5.5 μA/cm^2^, the sample at 443 K reduced from 9.9 to 4 μA/cm^2^, and the sample at 463 K reduced from 8.8 to 2.4 μA/cm^2^, as shown in Fig. 8b. Because I_corr_ is directly proportional to C.R. of the material, the decrease in I_corr_ reduces the C.R. (As illustrated in Fig. 8)^16,36^. Furthermore, there is a direct relationship between the addition of hard particles (CuO nanostructure, for instance) to the alloy and the grain size diameter, which is related to the improvement of the electrochemical and mechanical properties due to the uniform distribution of CuO nanostructure^20,37,38^. Raising the aging temperatures for both **A**_**1**_ and **A**_**2**_ reduces the corrosion rate.

Concerning the electrochemical corrosion tests, a small addition of nano CuO enhanced the surface area, reducing corrosion sites and slowing down the C.R. before and after aging^24^. The larger specific surface area surface of nano CuO may be responsible for its corrosion resistance. The aged samples appeared with a more uniform distribution of nano CuO in **A**_**1**_, which controls charge transfer in the composite surface regarding better corrosion rate during corrosion testing than **A**_**1**_**.** Accordingly, sample **A**_**2**_ containing nano CuO exhibits greater corrosion resistance than the other **A**_**1**_.

**Figure 9:**
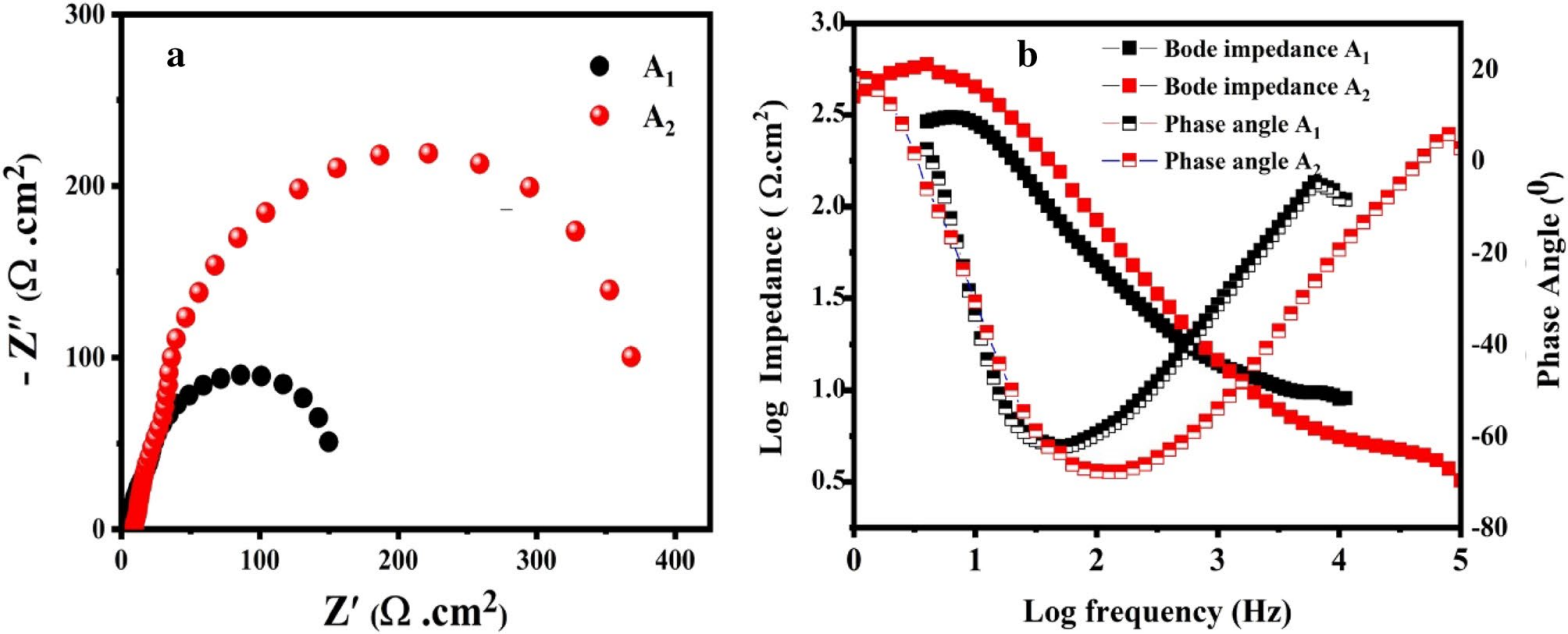
**(a)** Nyquist, (**b**) Bode impedance, and phase angle plots of **A**_**1**_ and **A**_**2**_ samples at aged temperature 463 K.

To obtain superior corrosion resistance both before and after aging, the **A**_**2**_ gives additional stability. EIS was utilized to describe the corrosion behavior of the **A**_**1**_ alloy and **A**_**2**_ in NaCl-based solution, as depicted in Fig. 9a. According to the findings, the **A**_**2**_ exhibits greater corrosion resistance than the **A**_**1**_. **A**_**2**_ may be well protected under corrosive conditions by the dense and stable surface, according to the greater phase angle and larger capacitive response, as shown in Fig. 9b. The phase angle and Bode impedance results match those from Nyquist plots. Because of this, the corrosion product layer on the** A**_**1**_ surface is less dense than on the **A**_**2**_ sample, which has caused **A**_**1**_ to corrode even more.

When **A**_**1**_ and **A**_**2**_ were aged (423, 443, and 463 K), corrosion resistance effectiveness was seen. The effect of nano CuO addition. This outcome was brought about by the condition of the possibility for pitting, cracks, and voids being minimal and smaller particle size. The blocking that turns the surface passive and shields the **A**_**2**_ surface from ionization and dissolution improved the nano sample’s corrosion resistance. This outcome demonstrates how the CuO addition enhances the Al–Zn surface’s ability to resist corrosion in water environments at room temperature^23^.

##### Morphology of the corroded surface

**Figure 10:**
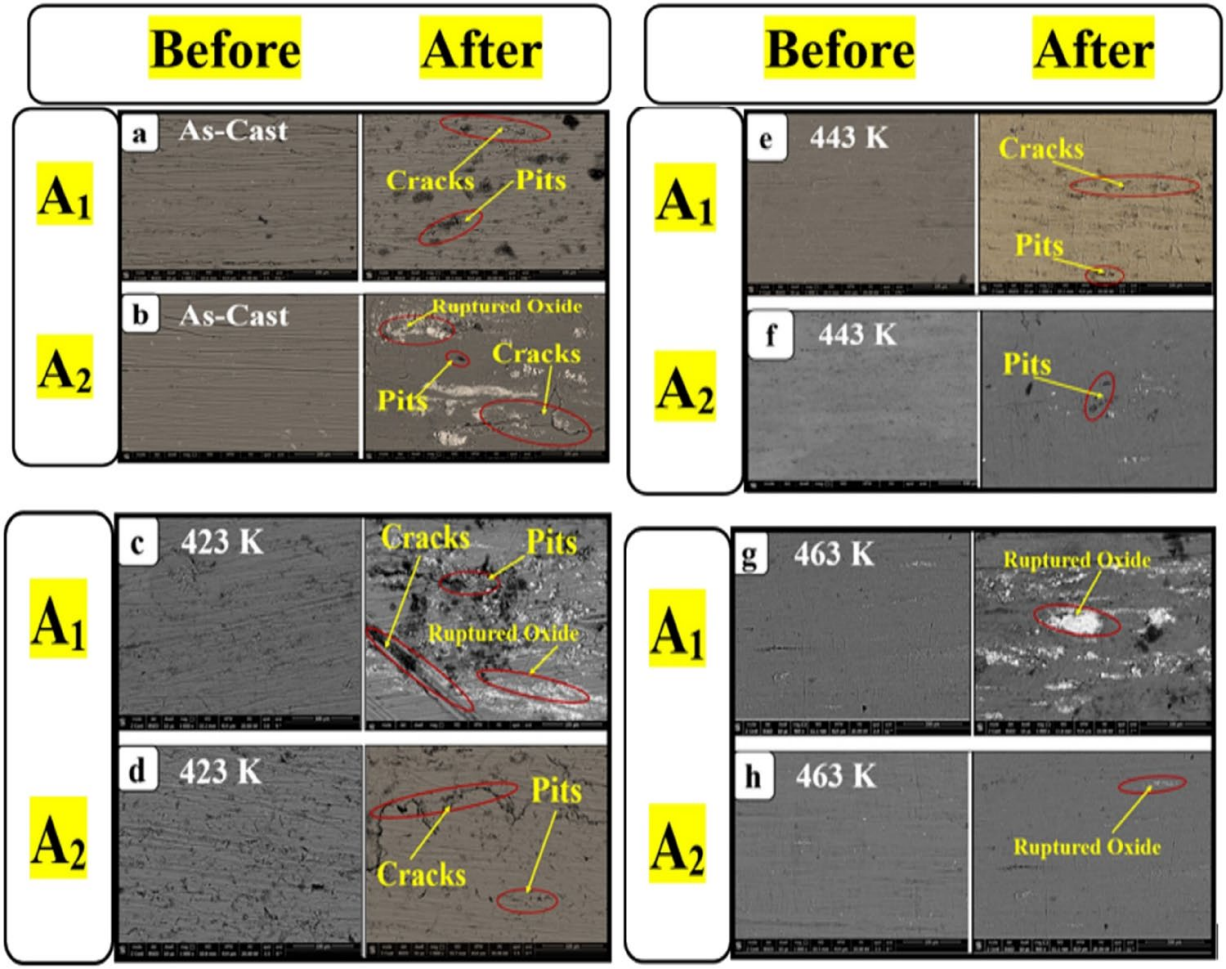
SEM images before and after corrosion test of the before and after different aging temperatures of **A**_**1**_ and **A**_**2**_ at (scale bar 100 μm).

The microstructure (SEM micrographs) for **A**_**1**_ and **A**_**2**_ samples are shown in Fig. 10, which were taken of the corroded surface before and after aging. Aging can change the sample surface microstructures, confirming the C.R. results. Figure 10a, c, e, g represent samples **A**_**1**_ and Fig. 10b, d, f, h represent sample** A**_**2**_. As shown in Fig. 10, the sample’s surfaces corroded by NaCl solution have irregular pitting, cracks, and ruptured oxide, which determine the sites of electrochemical activity of **A**_**1**_ and **A**_**2**_. As a result, **A**_**2**_ has a higher corrosion resistance than **A**_**1**_, which was very noticeable and confirmed the values of C.R. and I_corr_ for both samples, as shown in Fig. 8.

Before and after aging at 423 K, both **A**_**1**_ and **A**_**2**_ showed pits, cracks, and voids; however, above 423 K, nano CuO fractured into minute pieces, and at 443 K and 463 K, the nano CuO started to disappear. This finding is because Al–Zn alloy and CuO are different phases at first, causing incoherency between the two structures. During aging above 423 K, **A**_**2**_ became a single phase, significantly depleting the microcrack’s energy, reducing crack nucleation sites, and arresting its propagation ^39,40^.

Segregation is reduced when the grain size is smaller, and homogenous corrosion results. Improving grain refining lowers the corrosion rate^41–43^. A fine grain structure is more corrosion-resistant because a high grain boundary density encourages a superior oxide layer conduction rate on surfaces with low to passive corrosion rates. Several researchers have reported that dislocations have an impact on corrosion performance. These findings show that aging temperature has a significant and similar effect on the microstructure and electrochemical properties of the samples. As a result, it has been discovered that raising the aging temperature lowers the corrosion rate.

## Conclusions

The results of the study evidenced the following conclusions:

- The copper oxide was successfully prepared, and TEM analysis confirms it is in the nano-size range.
- Al–10Zn alloy and Al–10Zn–1CuO were successfully prepared and studied by XRD, SEM, and electrochemical measurements before and after different aging temperatures.
- Heat treatments offer an elegant way to modify the microstructure; as the aging temperature increased from 423 to 463 K, the addition of nano CuO caused grain refinement.
- OCP demonstrated that Al–10Zn–1CuO might be stable in 3.5% NaCl solution, nano 1CuO addition slowed down and shifted the potential to positive by increasing the temperature.
- It was found that polarization testing confirms the results of EIS, and microstructure (SEM & OP images), where Al–10Zn–1CuO revealed better corrosion resistance than Al–10Zn alloy in the 3.5% NaCl solution before and after aging, where the addition of nano CuO obstructed the defects (cracks, developing pits, and ruptured oxide).
- Aging has a significant influence on reducing the samples’ corrosion rate by increasing temperature.

## Data Availability

The datasets used and/or analyzed during the current study are available from the corresponding author upon reasonable request.
